# Native Structure-Based Peptides as Potential Protein–Protein Interaction Inhibitors of SARS-CoV-2 Spike Protein and Human ACE2 Receptor

**DOI:** 10.3390/molecules26082157

**Published:** 2021-04-09

**Authors:** Norbert Odolczyk, Ewa Marzec, Maria Winiewska-Szajewska, Jarosław Poznański, Piotr Zielenkiewicz

**Affiliations:** 1 Laboratory of Systems Biology, Institute of Experimental Plant Biology and Biotechnology, Faculty of Biology, University of Warsaw, Miecznikowa 1, 02-096 Warsaw, Poland; piotr@ibb.waw.pl; 2 Institute of Biochemistry and Biophysics, Polish Academy of Sciences, Pawińskiego 5a, 02-106 Warsaw, Poland; ewa.b@ibb.waw.pl (E.M.); mwin@ibb.waw.pl (M.W.-S.); jarek@ibb.waw.pl (J.P.)

**Keywords:** SARS-CoV-2, COVID-19, inhibitors of protein–protein interactions, peptides, drug design, coronavirus, angiotensin-converting enzyme-2, ACE2

## Abstract

Severe acute respiratory syndrome-coronavirus 2 (SARS-CoV-2) is a positive-strand RNA virus that causes severe respiratory syndrome in humans, which is now referred to as coronavirus disease 2019 (COVID-19). Since December 2019, the new pathogen has rapidly spread globally, with over 65 million cases reported to the beginning of December 2020, including over 1.5 million deaths. Unfortunately, currently, there is no specific and effective treatment for COVID-19. As SARS-CoV-2 relies on its spike proteins (S) to bind to a host cell-surface receptor angiotensin-converting enzyme-2(ACE2), and this interaction is proved to be responsible for entering a virus into host cells, it makes an ideal target for antiviral drug development. In this work, we design three very short peptides based on the ACE2 sequence/structure fragments, which may effectively bind to the receptor-binding domain (RBD) of S protein and may, in turn, disrupt the important virus-host protein–protein interactions, blocking early steps of SARS-CoV-2 infection. Two of our peptides bind to virus protein with affinity in nanomolar range, and as very short peptides have great potential for drug development.

## 1. Introduction

Infection caused by novel severe acute respiratory syndrome coronavirus 2 (SARS-CoV-2) is referred to as COVID-19 (coronavirus disease-2019) [1], and has become a global health problem within the first three months since its earliest incidence, noticed in December 2019 in China [2].One year later, at the beginning of December 2020, SARS-CoV-2 had infected over 65 million individuals worldwide and is responsible for over 1.5 million deaths [3].

Based on the genome sequence analysis, the new SARS-CoV-2 shows strong homology (over 79% of sequence identity) with another positive-sense, single-stranded RNA virus, severe acute respiratory syndrome-related coronavirus (SARS-CoV) [4], which was responsible for a global outbreak in 2003 [5]. This strong similarity quickly suggested that both viruses may share the same host cell receptor [4], which for SARS-CoV was well defined as angiotensin-converting enzyme-2 (ACE2) [6]. This hypothesis was further supported at the protein structural level, first by homology modeling [7], and then by experimental techniques; cryo-electron microscopy [8] and X-ray diffraction crystallography [9]. 

Indeed, SARS-CoV-2, in the early stages of its life cycle, utilizes cross-species protein–protein interactions for the host cell entry. This process is mediated by the receptor-binding domain (RBD) of the virus spike (S) glycoprotein [8], which directly interacts with human receptor ACE2, starting a cascade of events leading to penetration of the host cell membrane, and finally the virus replication [10]. Thus, disrupting the ACE2 and S proteins interaction may lead to blocking or significantly reducing the infection caused by the novel pathogen, and it is worth considering as a target for potential anti-SARS-CoV-2 therapy.

Peptides derived from native protein–protein interaction (PPI) sites of interacting partners are valuable starting points in developing effective inhibitors of protein–protein interactions, and have already been proven to be useful for the design of efficient PPI modulators [11,12]. As ACE2-S complex structures have been solved by experimental techniques, a rational starting point for the development of molecules disrupting the formation of protein–protein of the complex is available, and has already been utilized to propose several peptides against SARS-CoV [13,14], and even recently SARS-CoV-2 [15,16,17,18,19,20,21,22].

In the present study, we designed three short peptides, which are based on ACE2 structures as potential ligands of SARS-CoV-2 S protein. The peptides were synthesized and their binding was evaluated by microscale thermophoresis assay (MST). Two of these peptides bind to the RBD of S protein with a dissociation constant (K_D_) in nM range, and as very short peptides have great potential for further development.

## 2. Results

### 2.1. ACE2 Structure-Based Peptides Design

**Figure 1:**
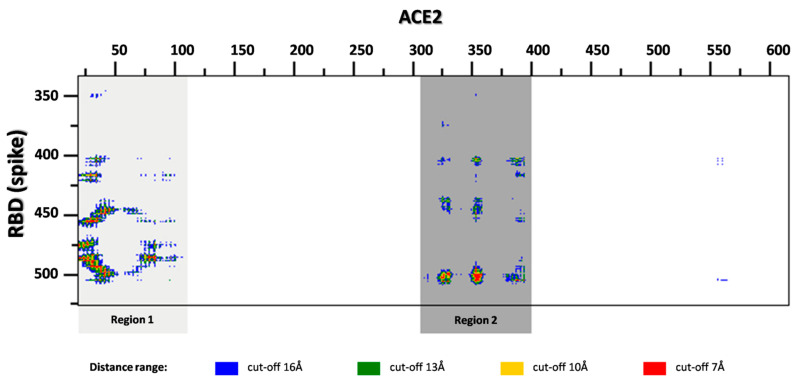
The distance range map of the inter-molecular contacts between the SARS-CoV-2 spike receptor-binding domain and human ACE2 (prepared by COCOMAPS server). Two regions in close contact have been highlighted by gray rectangles.

**Figure 2:**
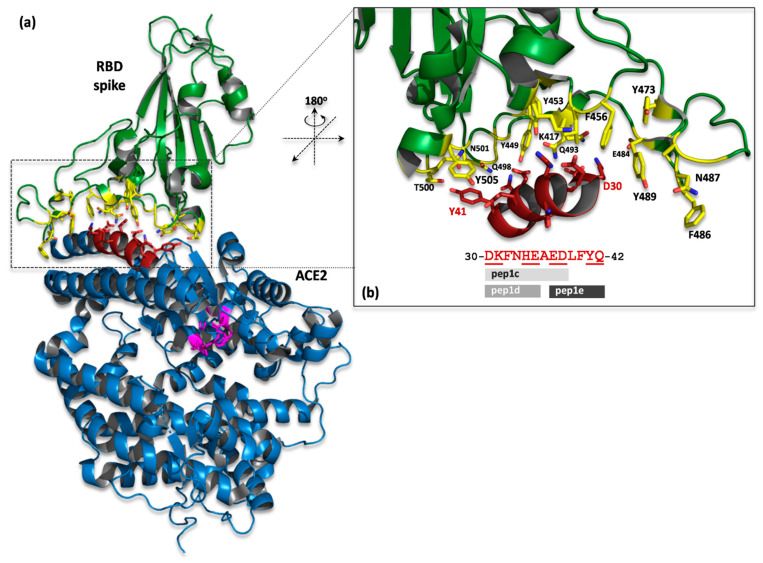
(**a**) Crystal structure of SARS-CoV-2 spike receptor-binding domain (green) bound with human ACE2 receptor (blue), the fragment of α1-helix selected as a prototype for peptide design is shown in red (PDB ID: 6M0J). The ACE2 catalytic motif (H345, P346, H374, E375, H378, and E402) is shown as magenta sticks. (**b**) Close up view of interacting interface between RBD and α-helical fragment D30-Q42 of ACE2. The crucial residues are presented as sticks (yellow/protein, red/peptide); Q493 is shown in two alternative positions. Prototype peptide sequence is also shown; residues responsible for interactions with protein are underlined.

Peptide ligands were designed by *in silico* analysis of the crystal structure (PDBid: 6M0J) of ACE2 receptor in complex with the RBD of S viral protein [23]. First, the interaction interfaces of both proteins have been defined according to the distance criteria (the cut-off distance was set to 8 Å) as implemented in the COCOMAPS (bioCOmplexesCOntact MAPS) software [24]. On the basis of the contact map, two regions of ACE2 have been identified on the interface (Figure 1). After visual inspection of the protein–protein complex, we focused our research on region 1, as it is located in the central part of the interface, with the single continuous sequence fragment containing the well-defined α-helical secondary structure, and creates mainly polar interactions (Figure 2) with S protein (especially D30-Q42 fragment). All these features make this region suitable for precursors of protein–protein interaction inhibitors [12]. Finally, three continuous sequence fragments of ACE2 protein, containing interaction interface epitopes from region 1, were selected as peptides: (pep1c) 30-DKFNHEAED-38, (pep1d) 30-DKFNHE-35, (pep1e) 37-EDLFYQ-42 (Figure 2).

The possible interactions of pep1d with S protein seem to be supported by two ion–ion contacts, D30:K417 and K31:E484, which are additionally stabilized by several hydrogen bonds created between D30:K417, K31:Q493, and E35:Q493 residues. In the pep1c, which is a longer version of pep1d, additional hydrogen bonds may occur between residues E37, D38 of the peptide and Y505, Y449 of the protein, respectively. In the case of pep1e, only one ion–ion interaction can be formed (between E37:R403), whereas hydrogen bonds may be created in pars E37:Y505, D38:Y449, Y41:T500, Y41:Q501, Q42:G446, Q42:Q498, and Q42:Y449.

In order to exclude the aggregation of native ACE2 peptides with ACE2 protein, which could interfere with its folding process [25,26], and as future drugs may possibly exhibit side effects, we introduced mutations into the designed peptides. All amino acid residues directly interacting with RBD were left unchanged, whereas on the other side of the interface, one position was modified in native sequence in each peptide as follows: pep1c and pep1d: F32G, and pep1e: L39G (Table 1). Proposed peptides were purchased and further subjected to experimental verification.

### 2.2. Binding of Peptides to RBD

**Figure 3:**
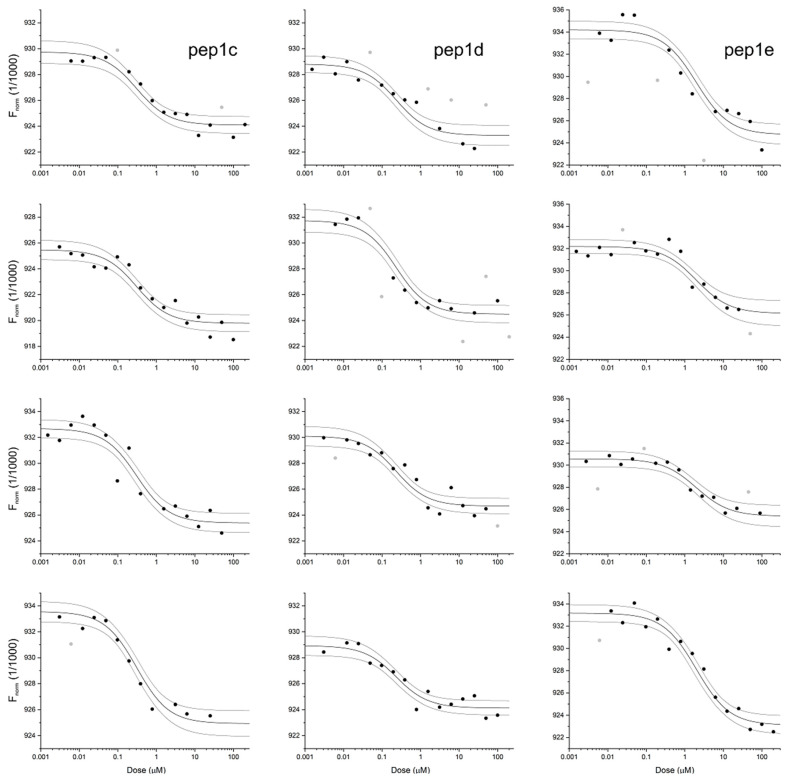
Interaction of RBD domain of SARS-CoV-2 protein with pep1c (**left**), pep1d (**center**) and pep1e (**right**), monitored with MST pseudo-titration experiments. Thick lines represent the model fitted for each peptide globally, using data from four independent experiments, while thin lines denote the 95% confidence bands for the fitted line. Gray circles identify data excluded from the analysis.

For three peptides, their binding affinity to the RBD domain of SARS-CoV-2 protein was estimated using microscale thermophoresis. For each of them, the four independent pseudo-titration experiments were found consistent (see Figure 3), so the globally fitted K_D_ values together with the accompanying standard errors were estimated. Pep1e displays moderate affinity to the RBD domain (K_D_ = 1.9 ± 0.4 μM), while pep1c and pep1d bind approximately 10-fold stronger (280 ± 60 nM and 210 ± 50 nM, respectively). As a negative control, we have tested interactions of RBD with MYEEFKAED peptide, which according to the proposed model of the complex displays a disrupted pattern of intermolecular interactions. MST-derived K_D_ of 0.55 ± 0.20 mM does identify this peptide as an extremely weak binder of the RBD (Figure S1 in Supplementary Material).

### 2.3. In Vitro RBD-ACE2 Interaction Inhibition Assay

The pep1c was tested in vitro for inhibition of the RBD-ACE2 complex formation. Inhibitory effect was monitored using the commercial COVID-19 Spike-ACE2 binding assay kit II (RayBiotech), as described in the Material and Methods section. The estimated IC_50_ for pep1c was 3.3 ± 0.8 mM (Figure S2 in Supplementary Material).

## 3. Discussion

As SARS-CoV-2 is a new pathogen, there is no specific treatment available today. However, immediately after the outbreak of SARS-CoV-2, the endeavor to develop a vaccine and a new targeted therapy against novel coronavirus has been started. Based on the sequence similarity to SARS-CoV, a few potential drug targets for future therapeutics have been proposed [27]. Most of such targets are non-structural enzymatic proteins (NSPs), which are crucial for virus replication and proliferation. However, therapeutics targeting the active site of particular virus enzymes may not be effective against future coronavirus epidemics, as virus mutations could make them futile. Thus, it is worth considering protein–protein interactions as potential drug targets, and we propose protein–protein interaction inhibitors as new antiviral therapeutics. As SARS-CoV-2 relies on its spike proteins (S) to bind a host cell-surface receptor ACE2, and this interaction is proved to be responsible for entering a virus into host cells, it makes an ideal target for antiviral drug development [28].

In this work, we have shown that two short hexapeptides, based on the ACE2 sequence/structure fragments, effectively bind to the RBD of S protein, and may in turn disrupt the important virus–host protein–protein interactions, blocking early steps of SARS-CoV-2 infection. However, we have additionally assessed the activity of pep1c against formation of the RBD–ACE2 complex. The estimated IC_50_ of 3.3 ± 0.8 mM is far too high for therapeutic applications; however, this peptide may state the starting point for further studies.

All of our peptides are based on the subsequence of the α1-helix ACE2 region [23], which have also been utilized in similar peptide-based strategies described elsewhere [15,16,17,18,19,20,21,22]. It is worthwhile to notice that peptides proposed by other groups are based on the assumption that larger parts of the interaction interface should be mimicked to retain strong binding affinity to S protein. Thus, while the shortest peptide sequence published till now, by Baig [16], contains 18 residues of ACE2, and the longest has been proposed by Han and Král [17], composed of 129 residues, both were proposed only by in silico methodology without experimental verification.

However, peptides are pharmaco-kinetically unfavorable for drug application, due to their low metabolic stability, lack of oral activity, rapid excretion, and low cell-membrane permeability [29]. Although the ACE2 is expressed in different tissues, lungs are in the first line of virus attachment. Thus, such presented short peptides can be administered directly via inhalation to this critical organ for SARS-CoV-2 infection, and could be an attractive alternative to the traditional drug development.

In conclusion, the results of this study have provided potential candidates for further development of therapeutics against SARS-CoV-2 infection. The possible scenarios worth considering for further drug development include: (i) optimization of our peptide sequences composition, (ii) design of peptidomimetic molecules, or (iii) to establish the competitive binding, high throughput screening assay, and utilize our short peptides to find non-peptidic, drug-like compounds as proposed elsewhere [30].

## 4. Materials and Methods

### 4.1. In Silico Design of Peptides

The ACE2-S complex structure (PDB ID: 6M0J) [23] was retrieved from the Protein Data Bank (www.rcsb.org, accessed on 28 April 2020) [31]. The protein–protein interaction interface was defined according to the method implemented in the bioCOmplexesCOntact MAPS server (COCOMAPS: https://www.molnac.unisa.it/Bio-Tools/cocomaps/, accessed on 6 May 2021) [24]. The analysis of the protein–protein interfaces and selection of ACE2fragments suitable for peptides were conducted on the basis of intermolecular contact maps created by COCOMAPS tool (the cut-off distance was set to 8 Å), and by visual inspection of the ACE2-S complex structure using PyMOL software (Version 2.3.0, Schrödinger, LLC, New York, NY, USA).

### 4.2. Domain RBD of SARS-CoV-2 Protein and ACE2-Based Peptides

The recombinant domain RBD of the SARS-CoV-2 subunit S1 (res Arg319—Phe541) was purchased from Genscript^®^/Raybiotech Inc. Nr cat. 230-30162 (declared purity > 95%). According to the supplier protocol, the domain was expressed in HEK293 cells.

Peptides were purchased from Genscript^®^ (declared purity: ≥95%).

### 4.3. Microscale Thermophoresis

All experiments were carried out with Monolith NT 115 (NanoTemper Technologies) apparatus using Premium MO-K025 capillaries. RBD domain carrying His6Tag was labeled with 2nd generation Monolith His-Tag Labeling Kit RED-tris-NTA fluorescence marker. The concentration of the protein was kept constant at 50 nM, while the concentration of ligands varied in the range of 0.5 nM–200 μM. For each peptide, the dissociation constant was estimated globally from the series of four independent pseudo-titration experiments based on the thermophoretic effect accompanying temperature jump as the reporter of protein–ligand interaction. The low- and high-concentration asymptotes were fitted individually for each experiment. All calculations were performed using the appropriate model [32,33] implemented in Origin 2020.

### 4.4. In Vitro RBD-ACE2 Interaction Inhibition Assay

The assay was performed with the SpectraMax iD3 Multi-Mode Microplate Reader (MolecularDevices), using a 96-well plate coated with recombinantly-expressed ACE2-COVID-19 Spike-ACE2 binding assay kit II (RayBiotech). The total amount of the bound recombinant S-RBD protein (with an Fc tag) in the presence of tested compounds was measured. For this purpose, the color reaction of HRP-conjugated IgG in the presence of 3,3′,5,5′-tetramethylbenzidine(TMB) substrate with the TMB solution, producing a blue color that is proportional to the amount of the bound fraction of S-RBD, was used. The HRP-TMB reaction was then halted with the addition of the Stop Solution, resulting in a blue-to-yellow color change. The experiment was performed according to manufacturer recommendation. To each well, 100 μLof tested compound in varying concentration with the recommended concentration of RBD protein was added, and the plate was incubated for 2.5 h at room temperature. After recommended washing, 100 μL of HRP-conjugated antibody solution was added and incubated for 1 h at room temperature. Next, after the same washing procedure, 100 μL of TMB One-Step Substrate Reagent was added and incubated for 30 min at room temperature in the dark. Reaction was stopped by adding 50 μL of Stop Solution to each well, and the resulting absorption at 450 nm was measured. Inhibitor concentration varied in the range of 10 μM–10 mM. The experiment was initially performed in technical duplicate (triangles in Figure S2 Supplementary Material), while the third repetition was conducted simultaneously for the separate sample preparation (diamonds in Figure S2 Supplementary Material). As a control, the fourth repetition was conducted using independent experimental procedure (circles in Figure S2 Supplementary Material). All of these results were analyzed globally using the sigmoidal dose-response equation implemented in the Origin 9.8 package. The IC_50_ value was defined commonly in all repetitions, while the high-concentration asymptote was taken for each series directly from the reference measurement for the same sample in the absence of S-RBD/pep1c (equivalent of the positive control). The low-concentration asymptotes were fitted individually for each experiment.

## Figures and Tables

**Table 1:** Sequences of ACE2 structure-based peptides.

Peptide	Sequence ^1^	K_D_ [nM] ^2^
pep1c	30-DKGNHEAED-38	280 ± 60
pep1d	30-DKGNHE-35	210 ± 50
pep1e	37-EDGFYQ-42	1900 ± 400

^1^ The amino acid residues that have been modified according to the original ACE2 sequence (F32G for pep1c and pep1d; L39G for pep1e) are underlined. ^2^ The dissociation constant estimated globally from the series of four independent pseudo-titration experiments from MST.

## Data Availability

The data presented in this study are available on request from the corresponding author.

## Supplementary Materials

The following are available online, Figure S1: Interaction of RBD domain of SARS-CoV-2 protein with MYEEFKAED peptide monitored with MST pseudo-titration experiments, Figure S2. The RBD-ACE2 interaction inhibition assay.

## Sample Availability

Not available.
