# Supplementary material for: Native Structure-Based Peptides as Potential Protein–Protein Interaction Inhibitors of SARS-CoV-2 Spike Protein and Human ACE2 Receptor
Source: Molecules. 2021 Apr 9;26(8):2157. doi: 10.3390/molecules26082157 (PMC8070189; doi:10.3390/molecules26082157)
Supplement: Supplementary file 1 [file molecules-26-02157-s001.pdf]

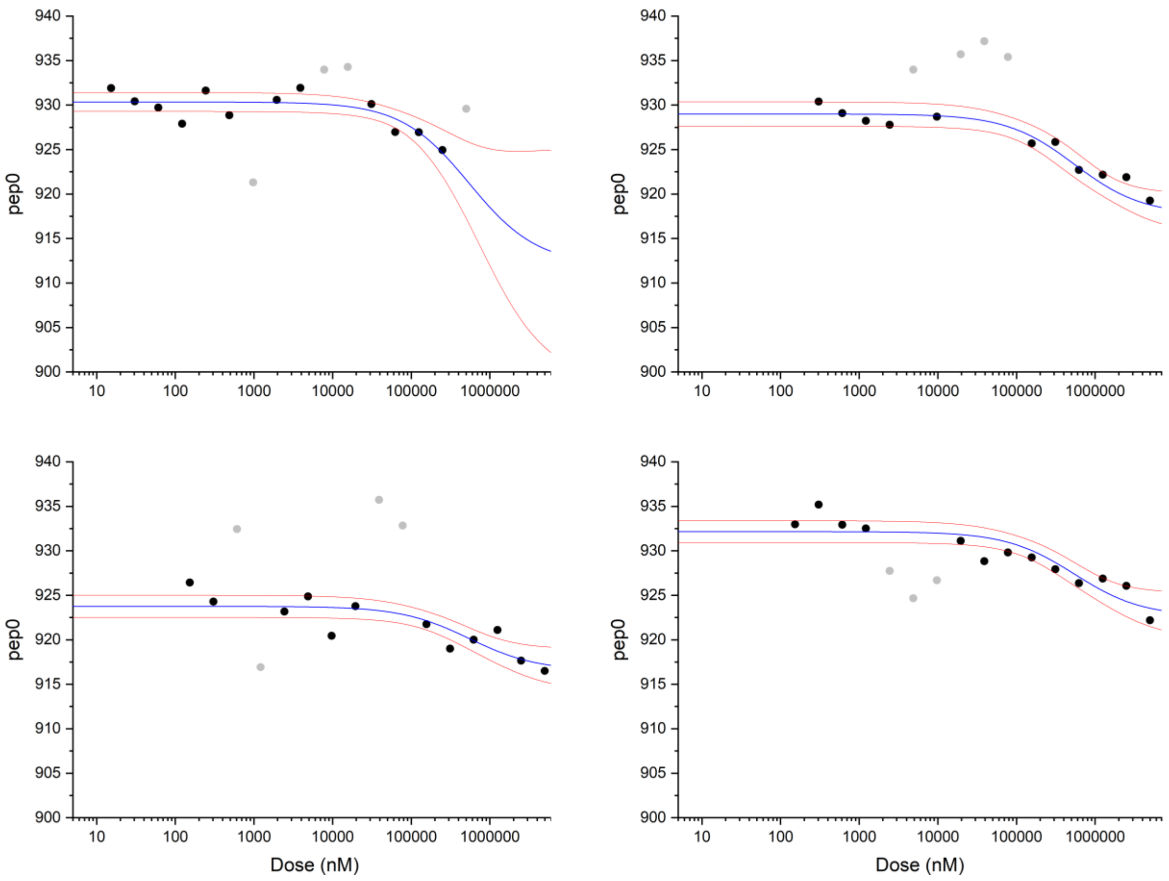

Figure S1. Interaction of RBD domain of SARS-CoV-2 protein with MYEEFKAED peptide, (which according to the proposed model of the complex displays a disrupted pattern of intermolecular interactions) monitored with MST pseudo-titration experiments. Blue lines represent the model fitted for each peptide globally using data from four independent experiments, while red lines denote the 95% confidence bands for the fitted line. Gray circles identify data excluded from the analysis.

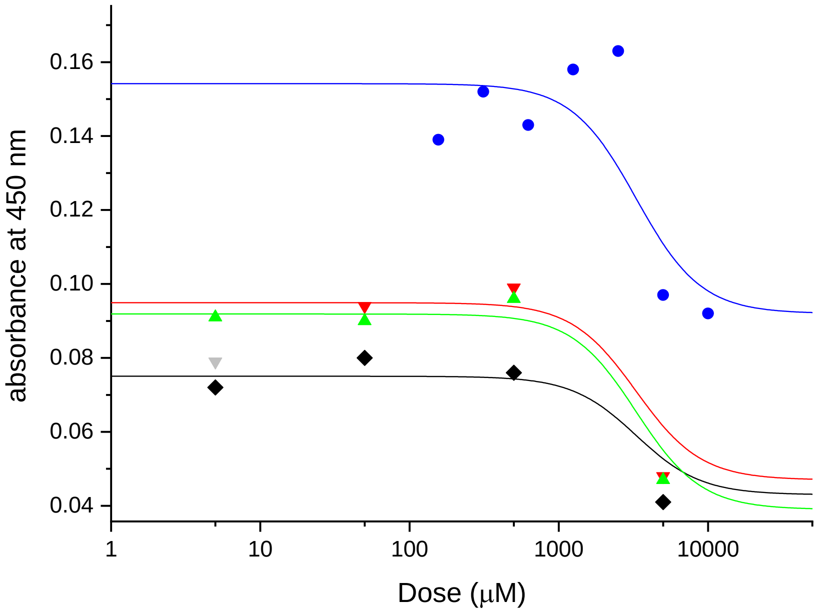

Figure S2. The RBD-ACE2 interaction inhibition assay. Inhibitor concentration varied in the range of 10μM-10mM. The experiment was initially performed in technical duplicate (triangles), while the third repetition was done simultaneously for the separate sample preparation (diamonds). As a control, the fourth repetition was done using independent experimental procedure (circles). All these results were analyzed globally using the sigmoidal dose-response equation and the IC_{50} value was defined common in all repetitions.
